# A comparative analysis of inequalities in sexual violence before age 18 among women in Ghana using the WHO health equity assessment toolkit, 2008 and 2022

**DOI:** 10.3389/fpsyt.2026.1816366

**Published:** 2026-07-29

**Authors:** Amidu Alhassan, Yula Salifu, Abraham Norman Nortey, Mustapha Amoadu, Patience Fakornam Doe, Augustus Osborne, Joseph Lasong

**Affiliations:** 1 Department of Adult Health, School of Nursing and Midwifery, University of Cape Coast, Cape Coast, Ghana; 2 Department of Nursing, All Nations University, Koforidua, Ghana; 3 Department of Population, Family and Reproductive Health, University of Ghana, Accra, Ghana; 4 Department of Population and Reproductive Health, University for Development Studies, Tamale, Ghana; 5 Department of Community Medicine, University of Cape Coast, Cape Coast, Ghana; 6 Biomedical and Clinical Research Centre, University of Cape Coast, Cape Coast, Ghana; 7 Department of Public Health, University of Cape Coast, Cape Coast, Ghana; 8 Institute for Development, Freetown, Sierra Leone

**Keywords:** childhood abuse, Ghana, inequalities, sexual violence, WHO HEAT

## Abstract

**Background:**

Sexual violence before age 18 is a severe public health concern with long-lasting consequences. Despite Ghana’s legal and policy reforms including the Domestic Violence Act of 2007, differences in the burden of childhood sexual violence across population subgroups remain inadequately characterised. This study examined differences in the estimated prevalence of childhood sexual violence among young Ghanaian women between 2008 and 2022, with a specific focus on age cohort and marital-status subgroups.

**Methods:**

A secondary comparative analysis of the 2008 and 2022 Ghana Demographic and Health Surveys was conducted using the WHO Health Equity Assessment Toolkit. The primary outcome was the self-reported proportion of women who experienced sexual violence before age 18. Summary inequality measures included absolute difference, ratio, population-attributable fraction (PAF), and population-attributable risk (PAR), with 95% confidence intervals.

**Results:**

The estimated national prevalence of childhood sexual violence was lower in 2022 (6.4%) than in 2008 (8.3%). Never-married women had the highest estimated prevalence in both years (11.3% in 2008; 8.4% in 2022). Among age cohorts, women aged 20–24 years had the highest estimate in 2008 (10.5%), declining to 7.0% in 2022. However, estimates increased among women aged 35–39 (5.1% to 6.6%) and 40–44 years (3.4% to 5.1%), suggesting a cohort-specific burden pattern. The age-related PAF shifted from −9.3% (95% CI: −9.8 to −8.9) in 2008 to −44.2% (95% CI: −44.4 to −43.9) in 2022, reflecting a more protective age distribution. The age PAR became statistically significant, moving from −0.7% (95% CI: −3.8 to 2.5) to −2.5% (95% CI: −4.0 to −1.0). Marital-status differences showed modest differences: PAF from −18.0% to −16.4%, and PAR from −1.5% to −1.1% (CI crossing zero in 2022).

**Conclusions:**

The estimated prevalence of childhood sexual violence was lower in 2022 than in 2008 across most subgroups; however, differences in burden by age cohort and marital status persisted, with never-married women and some older cohorts carrying a disproportionate burden. These patterns highlight the need for equity-targeted prevention strategies and intensified support for never-married young women.

## 1 Introduction

Sexual violence experienced before the age of 18 represents one of the most severe human rights violations and public health challenges affecting women globally. A landmark systematic analysis published in The Lancet pooled data from 204 countries and estimated that globally 12.7% of women had experienced sexual violence before age 18, with the burden substantially higher in sub-Saharan Africa, approximately 18.6%, than in most other regions (1). The World Health Organization (WHO) estimates that one in three women worldwide experiences sexual violence at some point in their lifetime (2). Among girls and adolescent women, the consequences of early sexual victimisation extend across the life course, affecting educational attainment, economic participation, mental health, and reproductive health outcomes (3).

In sub-Saharan Africa, childhood sexual violence occurs within complex social, economic, and legal contexts characterised by gender inequality, patriarchal norms, limited legal enforcement, and inadequate survivor support services (4, 5). Evidence demonstrates that never-married adolescent girls, those with limited education, and those in rural communities face disproportionately high risks, often compounded by barriers to disclosure rooted in stigma, fear of reprisal, and community pressure to protect family honour (6, 7). Despite growing global attention, the epidemiological evidence in Ghana remains fragmented, with national DHS reports providing aggregate prevalence figures but seldom disaggregating findings by equity-relevant dimensions such as age cohort or marital status (8, 9).

In Ghana, one in seven women (approximately 14%) aged 15–49 years has experienced sexual violence at some point in her life, according to the 2022 Ghana Demographic and Health Survey (10). Among women aged 18–29 years specifically, the estimated prevalence of sexual violence before age 18 was 8.3% in 2008 and 6.4% in 2022 (10, 11). These figures, while reflecting lower estimates in the more recent survey, mask important heterogeneity across population subgroups and must be contextualised within a broader pattern of under-reporting driven by stigma and inadequate legal enforcement (12).

The consequences of childhood sexual violence for Ghanaian women are well-documented and severe. Evidence consistently links early sexual victimisation to depression and depressive disorders (13, 14), anxiety and post-traumatic stress (15, 16), sexually transmitted infections including HIV (17), and compromised social functioning (18, 19). Ahinkorah (20) demonstrated that in West Africa, adolescent girls who experienced sexual violence had significantly worse reproductive health outcomes and were more likely to experience early childbearing and intimate partner violence in adulthood (20). In Ghana specifically, Tenkorang (21) found that sexual violence at first intercourse was associated with increased risk of subsequent sexual coercion and lower contraceptive use (21). These findings underscore the urgency of population-level surveillance and equity analysis.

Additionally, Ghana’s legal and policy environment for addressing gender-based violence underwent significant development between the two survey rounds examined in this study. The Domestic Violence Act of 2007 established a legal framework for prosecuting domestic and sexual violence, and the Domestic Violence and Victim Support Unit (DOVVSU) was mandated to respond to survivors (22). Despite these reforms, enforcement remains uneven, particularly in rural areas, where traditional authorities may supersede formal legal systems, and where DOVVSU presence is limited (23). The National Gender Policy (revised 2015) and Ghana’s ratification of the African Charter on the Rights and Welfare of the Child have further strengthened normative commitments, yet implementation gaps persist.

Further, cultural norms in Ghana frequently discourage disclosure of sexual violence: societal prioritisation of family honour, fear of stigmatisation, and economic dependence on perpetrators or their families all contribute to significant under-reporting (24, 25). Boakye et al. (26) analysed case records of child sexual abuse in Ghana and found that community-level disclosure patterns were heavily shaped by the relationship between victim and perpetrator and by the perceived threat to family reputation (26). Lu et al. (27) further documented that law enforcement responses to childhood sexual abuse remained inadequate, with low prosecution rates and limited victim support (27). These structural and socio-cultural realities mean that observed differences in survey estimates between 2008 and 2022 may partially reflect differences in disclosure behaviour and survey methodology rather than only true differences in exposure.

Studies using the HEAT framework in Ghana and the wider sub-Saharan African region have examined inequalities in child nutrition, immunisation coverage, and maternal health service utilisation, consistently demonstrating the analytical utility of HEAT for identifying persistently disadvantaged subgroups and tracking the distribution of health burdens over time (28–31). However, to our knowledge, no published study has applied the WHO HEAT platform to examine differences in the burden of childhood sexual violence in Ghana or in sub-Saharan Africa more broadly. This represents a critical evidence gap, given that HEAT’s standardised summary measures allow the magnitude and distribution of disparities to be characterised in a way that simple national prevalence estimates cannot.

The period between 2008 and 2022 represents a substantive window of policy transition in Ghana’s response to gender-based violence. The 2008 GDHS was conducted immediately following the enactment of the Domestic Violence Act, while the 2022 GDHS captured a population that had lived under strengthened legal and policy frameworks for 14 years. Understanding whether, and how equitably, the burden of childhood sexual violence differs between these two time points is essential for accountability and for evidence-based programme design. Despite national DHS data being available, no study had used the WHO HEAT framework to quantify how the burden of childhood sexual violence before age 18 was distributed across age cohorts and marital-status groups in Ghana across these two rounds. Existing analyses report national prevalence only, without characterising disparities or how they have evolved between these two surveys.

To our knowledge, this is the first study to apply the WHO HEAT framework to examine differences in the estimated prevalence of sexual violence before age 18 among young Ghanaian women across age cohorts and marital-status groups, comparing two nationally representative survey rounds spanning 14 years of significant policy reform (2008–2022).

Thus, this study aims to: (i) compare the estimated prevalence of childhood sexual violence among women aged 18–29 years in Ghana between 2008 and 2022; (ii) quantify and compare differences in burden across age cohorts and marital-status groups using HEAT summary inequality measures; and (iii) identify subgroups that carry a disproportionate burden and where disparities have not been adequately reduced over this period.

## 2 Methods

### 2.1 Study design and data sources

This study employed a secondary comparative analysis of nationally representative survey data from Ghana using the WHO HEAT software (version 5.0). The design was cross-sectional and comparative across two time points. Data were drawn from the 2008 and 2022 Ghana Demographic and Health Surveys (GDHS), accessed through the WHO HEAT platform. Both datasets provide anonymised, population-based estimates that are weighted to be nationally representative and are suitable for equity-focused analyses. The DHS is a globally standardised household survey programme conducted approximately every five years in Ghana, collecting data on health, nutrition, and population dynamics from women of reproductive age (15–49 years) (32). The decision to compare 2008 and 2022 was determined by: (a) the availability of the childhood sexual violence indicator in both rounds within the HEAT platform, and (b) the policy significance of this interval, spanning the introduction of Ghana’s Domestic Violence Act (2007) to the most recent available survey round.

Regarding the omission of the 2014 GDHS: The 2014 GDHS round was considered for inclusion. However, disaggregated estimates for childhood sexual violence before age 18 among women aged 18–29 years were not reliably available within the HEAT platform for that round at the time of analysis. We acknowledge that HEAT Plus permits users to upload their own estimates. However, while technically feasible, this would require independent calculation of 2014 subgroup estimates from individual-level DHS data, which falls outside the scope of the present study’s methodology, which intentionally leverages the HEAT platform’s standardised, pre-loaded estimates to ensure reproducibility and alignment with WHO reporting frameworks. Thus further analysis should (a) prepare the 2014 GDHS estimates and upload them using HEAT Plus, or (b) request WHO to have the 2014 data included in the WHO Health Inequality Data Repository. This will enable a three-round comparative analysis (2008, 2014, 2022) in future work. For the present study, the comparison of 2008 and 2022 represents the two time points with the greatest policy relevance (immediately post-Domestic Violence Act and most recent available data) and is presented transparently as a two-point comparative analysis, not as a multi-point trend.

### 2.2 Study population

The reference population for national prevalence estimates comprised women aged 18–29 years as provided in the WHO HEAT country summary profile for Ghana. This age group was selected because: (a) all women aged 18–29 had necessarily already passed through the exposure window of interest (childhood and adolescence, defined as before age 18); (b) this cohort reflects the most recent exposures to Ghana’s evolving child protection environment; and (c) restricting to younger adult women reduces confounding from cohort-specific disclosure patterns that may affect older women’s retrospective reporting of childhood events.

For the age-stratified subgroup analysis, the WHO HEAT platform provides disaggregated estimates in standardised five-year age bands spanning 20–49 years (specifically: 20–24, 25–29, 30–34, 35–39, 40–44, and 45–49 years). These bands are determined by the HEAT platform’s data structure and represent a constraint of the HEAT interface rather than a design choice. The age-stratified subgroup analysis therefore extends beyond the 18–29-year reference population to encompass older cohorts (30–49 years), providing a generational perspective on how the burden of childhood sexual violence differs across women who spent their childhoods under different historical conditions. This distinction between the reference population for national prevalence (18–29 years) and the age-stratified subgroup analysis (20–49 years) is clearly maintained throughout the manuscript and represents a platform-determined analytical constraint, which is acknowledged as a limitation.

Age and marital status were selected as the primary stratifying dimensions in this analysis for both substantive and data-availability reasons. Age captures generational differences in exposure to Ghana’s evolving child protection systems, with younger cohorts having experienced childhood under stronger legal protections than older women. Marital status is a critical social determinant of sexual violence risk, reflecting power dynamics within partnerships, levels of social support and autonomy, and structural vulnerability to coercive relationships (33, 34). Although other stratifiers including household wealth, educational attainment, place of residence, and geographic region, are conceptually important, disaggregated data for childhood sexual violence across these dimensions were not available in the HEAT platform, and their exclusion is acknowledged as a limitation.

The analytical distinction between the reference population for national prevalence estimates (women aged 18–29 years) and the population for age-stratified subgroup analyses (women aged 20–49 years) warrants explicit justification. The reference population of 18–29 years was selected because it represents the standard WHO HEAT country summary profile for Ghana, enabling direct comparison of national prevalence between survey rounds. This age range ensures that all respondents have passed through the entire childhood exposure window (under age 18) while minimising differential recall bias that may affect older cohorts.

In contrast, the age-stratified subgroup analysis uses the pre-defined disaggregation bands embedded in the WHO HEAT platform, which are standardised five-year intervals from 20–24 years up to 45–49 years. This platform-determined structure unfortunately excludes women aged 18–19 years from the stratified analysis, a limitation we explicitly acknowledge. The exclusion of this youngest cohort is important because these women have the most recent recall of childhood experiences, and their absence may under- or overestimate disparities if their experiences differ systematically from those aged 20–24 years.

The inclusion of older cohorts (30–49 years) in the age-stratified analysis, while not directly comparable to the 18–29-year reference population, provides valuable generational insights. These older women spent their childhoods under different historical conditions, specifically the 1980s and 1990s, when Ghana lacked comprehensive child protection laws. Comparing their reported prevalence to that of younger cohorts reveals how the burden of childhood sexual violence has differed across generations, even after accounting for potential recall and disclosure differences.

Regarding the feasibility of alternative analyses using original DHS datasets: such an approach would indeed allow a unified age definition (e.g., 18–49 years in five-year bands) and is methodologically desirable. However, this study was intentionally designed to use the WHO HEAT platform to leverage its standardised, reproducible summary measures (D, R, PAF, PAR) that align with WHO equity monitoring frameworks and facilitate cross-country comparability. Accessing individual-level DHS data would require a separate data application process and different analytical software; while feasible, it falls outside the scope of the present study. Future research should pursue individual-level analyses to validate and extend our findings using a consistent age definition across all stratifiers.

### 2.3 Outcome measure

The primary outcome was the self-reported proportion of women who experienced sexual violence before age 18 (expressed as a percentage). In the GDHS, this is operationalised through a module asking women to report whether they experienced any form of sexual violence (including rape, sexual coercion, and unwanted sexual touching) before their 18th birthday. Estimates for each subgroup and for the national reference population were extracted from the HEAT platform for both 2008 and 2022.

### 2.4 Equity stratifiers

Two inequality dimensions were examined: (1) age group (20–49 years, based on the disaggregation categories available in the HEAT platform) and (2) marital status (married or living together; never married; widowed, divorced, or separated). These stratifiers were selected because they represent key social determinants of both exposure to childhood sexual violence and patterns of disclosure and reporting. Age captures generational cohort effects and exposure to different child protection environments. Marital status reflects social power dynamics, community oversight, and structural vulnerability. Although other relevant dimensions, including household wealth, educational attainment, place of residence, and geographic region, are conceptually important determinants of childhood sexual violence risk, they were not available as disaggregated stratifiers for this indicator within the HEAT platform. Their exclusion is acknowledged as a limitation and is explicitly discussed.

### 2.5 Summary inequality measures

The WHO HEAT platform computed four complementary summary measures of disparity, each capturing a distinct dimension of between-group differences. These measures are described below, with their mathematical formulations provided for methodological transparency:

Absolute difference (D): The simple arithmetic difference in estimated prevalence between the subgroup with the highest and the subgroup with the lowest prevalence: $\text{D}={\text{P}}_{\max}-{\text{P}}_{\text{m}}^{{\text{I}}_{\text{n}}}$. A positive value indicates that the highest-prevalence subgroup experiences a greater absolute burden. Confidence intervals are not computed for D in the HEAT platform when sample size adjustments are embedded in the estimates.

Ratio (R): The relative difference, calculated as the ratio of the highest to the lowest subgroup prevalence: $\text{R}={\text{P}}_{\max}/{\text{P}}_{\text{m}}^{{\text{I}}_{\text{n}}}$. An R greater than 1.0 indicates that the highest-burden subgroup experiences a proportionally greater prevalence. Confidence intervals are not computed for R in the HEAT platform.

Population-attributable fraction (PAF): The proportional reduction in national prevalence that would be observed if all subgroups attained the prevalence of the reference (most favourable, lowest-prevalence) group: $\text{PAF}=\sum_{\text{i}}\left[{\text{w}}_{\text{i}}\left({\text{P}}_{\text{i}}-{\text{P}}^{\text{ref}}\right)\right]/{\text{P}}^{\text{nat}}$, where w_i_ is the population weight of subgroup i, P_i_ is the prevalence in subgroup i, P^ref^ is the prevalence in the reference (best-performing) subgroup, and P^nat^ is the national prevalence. A negative PAF indicates that, if disparities were eliminated and all groups attained the prevalence of the most favourable group, national prevalence would fall. More negative values indicate a greater potential benefit from equity-targeted intervention. 95% CIs are computed for PAF.

Population-attributable risk (PAR): The absolute reduction in national prevalence achievable if all subgroups attained the reference group’s prevalence: PAR = P^nat^ − P^ref^. Equivalently, PAR = PAF × P^nat^/100. A more negative PAR indicates a larger absolute gain from eliminating between-group disparities. 95% CIs are computed for PAR; CIs that cross zero indicate that the estimated absolute gain is not statistically distinguishable from zero at the 5% significance level.

All four measures were computed for both 2008 and 2022, enabling a comparative characterisation of how the magnitude and distribution of disparities differed between the two survey rounds. For PAF, values of 5–10% (in absolute terms) are considered indicative of small disparities, 10–20% moderate, and >20% large, following established thresholds in health inequality literature.

HEAT is used here as a descriptive and comparative analytical tool. It quantifies and compares subgroup differences using standardised summary measures but does not model causal pathways, control for confounders, or support causal inference. All findings are interpreted in descriptive terms, and the analytical limitations of this approach are explicitly acknowledged.

### 2.6 Ethical considerations

This study used publicly available, anonymised secondary data from the Ghana Demographic and Health Surveys, accessed via the WHO HEAT platform. Individual-level data were not accessed. Ethical approval was therefore not required. The analysis adhered to ethical principles of respect for survivors by employing non-stigmatising language, focusing on systemic rather than individual-level factors, and maintaining strict confidentiality as embedded in the original survey design. The research aligns with the WHO’s standards for equity-oriented assessments.

## 3 Results

### 3.1 National prevalence of childhood sexual violence among women aged 18–29 years

**Figure 1:**
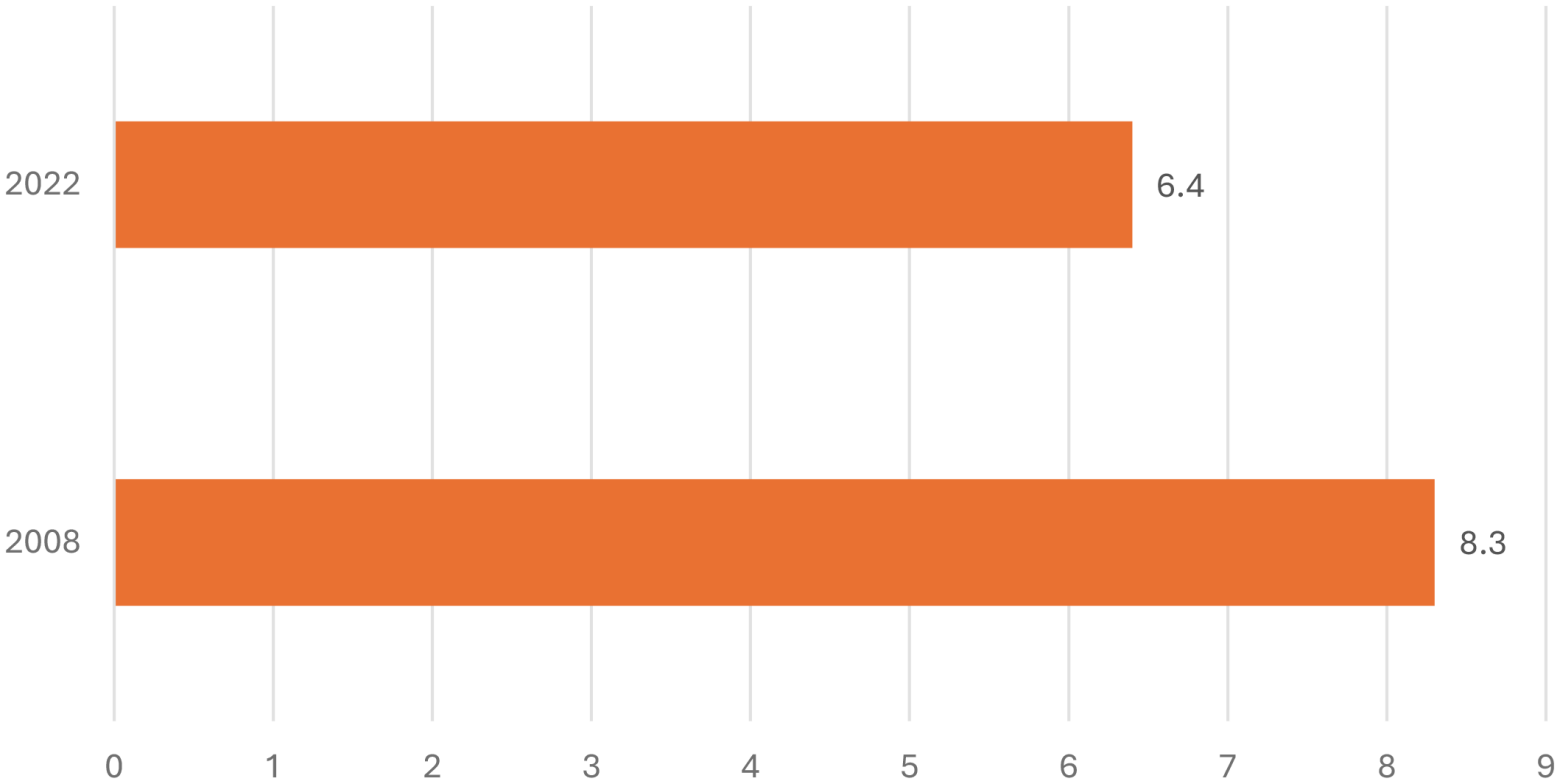
Estimated national prevalence of sexual violence before age 18 among women aged 18–29 years, Ghana, 2008 and 2022.

The estimated national prevalence of sexual violence before age 18 among women aged 18–29 years in Ghana was 8.3% in 2008 and 6.4% in 2022 (**Figure 1**). This represents an absolute difference of 1.9 percentage points between the two survey rounds. It is important to note, however, that this comparison reflects two cross-sectional estimates from population surveys conducted 14 years apart. The observed lower estimate in 2022 may reflect a genuine difference in the prevalence of childhood sexual violence, differences in disclosure norms and willingness to report such experiences in surveys, improvements in survivor awareness, or differences in survey methodology between rounds.

### 3.2 Differences in childhood sexual violence burden by marital status

**Figure 2:**
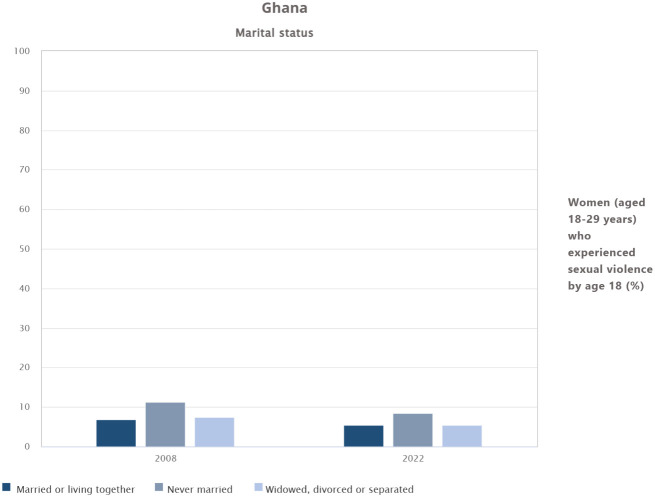
Estimated prevalence of childhood sexual violence by marital status, Ghana, 2008 and 2022.

Among marital-status subgroups, never-married women had the highest estimated prevalence in both survey years: 11.3% in 2008 and 8.4% in 2022. Women who were married or living together had estimates of 6.8% (2008) and 5.5% (2022), while those who were widowed, divorced, or separated recorded 7.5% (2008) and 5.4% (2022) (**Figure 2**). Across all three marital-status groups, the estimated prevalence was lower in 2022 than in 2008.

Never-married women thus maintained the highest burden in both years, with an absolute difference from the most favourable group (widowed/divorced/separated in 2022) of 3.0 percentage points in 2022, compared with 4.5 percentage points in 2008 (**Table 1**). The ratio of the most to least affected group was 1.7 in 2008 and 1.6 in 2022, indicating only a modest difference in relative disparity. The PAF changed from −18.0% (95% CI: −18.1 to −17.9) in 2008 to −16.4% (95% CI: −16.6 to −16.1) in 2022; both estimates are large in magnitude (>10% in absolute terms) and have CIs that exclude zero, confirming the statistical robustness of the marital-status disparity in both years. The PAR shifted from −1.5% (95% CI: −2.4 to −0.6) in 2008 to −1.1% (95% CI: −2.8 to 0.7) in 2022. Notably, the 95% CI for the 2022 PAR crosses zero, indicating that the absolute contribution of marital-status disparities to national prevalence was not statistically significant at the 5% level in 2022, reflecting the narrowing of between-group absolute differences.

**Table 1:** Summary measures of differences in childhood sexual violence burden across age and marital-status subgroups, in Ghana, 2008 and 2022.

		2008			2022				
Dimension	Measure	Est. (%)	95% CI-LB	95% CI-UB	Est. (%)	95% CI-LB	95% CI-UB	Change 2008→2022	Sig.
Age (20–49 yrs)	D	4.1	NA	NA	3.8	NA	NA	−0.3	
	R	1.6	NA	NA	2.2	NA	NA	+0.6	
	PAF (%)	−9.3	−9.8	−8.9	−44.2	−44.4	−43.9	−34.9	***
	PAR (%)	−0.7	−3.8	2.5	−2.5	−4.0	−1.0	−1.8	*
Marital status	D	4.5	NA	NA	3.0	NA	NA	−1.5	
	R	1.7	NA	NA	1.6	NA	NA	−0.1	
	PAF (%)	−18.0	−18.1	−17.9	−16.4	−16.6	−16.1	+1.6	***
	PAR (%)	−1.5	−2.4	−0.6	−1.1	−2.8	0.7	+0.4	ns

D, absolute difference (highest minus lowest subgroup prevalence); R, ratio (highest divided by lowest subgroup prevalence); PAF, population-attributable fraction; PAR, population-attributable risk; CI-LB/UB, 95% confidence interval lower/upper bound; NA, not available (confidence intervals not computed for D and R in the HEAT platform); ns, not statistically significant (95% CI crosses zero); * p< 0.05 (CI excludes zero); *** both 2008 and 2022 estimates have CIs excluding zero. Change column reports the arithmetic difference in the summary measure between 2008 and 2022 (2022 minus 2008).

### 3.3 Differences in childhood sexual violence burden by age cohort

**Figure 3:**
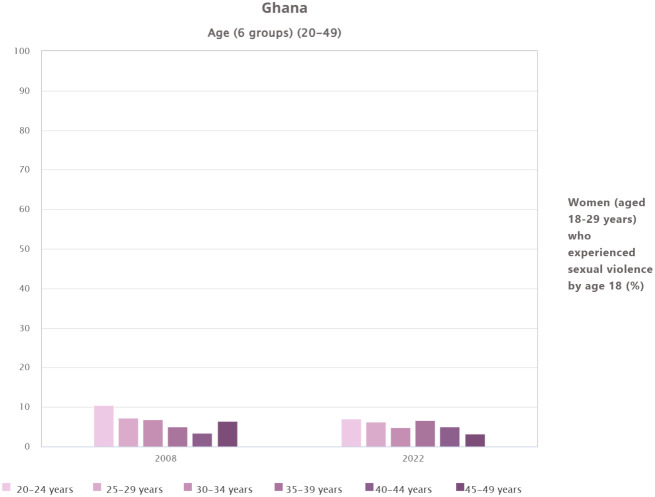
Estimated prevalence of childhood sexual violence by age group (20–49 years), Ghana, 2008 and 2022.

Among age cohorts, women aged 20–24 years had the highest estimated prevalence in 2008 (10.5%), followed by those aged 25–29 years (7.2%) and 30–34 years (6.9%). By 2022, estimates for these groups had declined to 7.0%, 6.2%, and 4.9%, respectively (**Figure 3**). However, the pattern was not uniform across all age groups: estimates increased among women aged 35–39 years (from 5.1% in 2008 to 6.6% in 2022) and those aged 40–44 years (from 3.4% to 5.1%). The sharpest reduction occurred among women aged 45–49 years (from 6.4% to 3.2%).

These cohort-specific patterns suggest a complex generational picture. Younger age groups (20–34 years), who experienced their childhoods under progressively stronger child protection frameworks, showed lower estimates in 2022 than in 2008. In contrast, the increases among women aged 35–44 years in 2022 may reflect differences in cohort-specific disclosure behaviour, changing willingness to report historical victimisation, or genuine age-period-cohort effects that cannot be fully disentangled from a two-round cross-sectional comparison.

### 3.4 Summary inequality measures of age and marital status dimensions

**Table 1** presents the full suite of HEAT summary inequality measures for both dimensions and both years. For the age dimension, the absolute difference between the highest and lowest age group declined from 4.1 percentage points in 2008 to 3.8 percentage points in 2022, and the ratio increased from 1.6 to 2.2, indicating that while absolute disparity narrowed slightly, the relative difference widened as the lowest-prevalence group (45–49 years in 2022) declined more steeply. The PAF shifted markedly from −9.3% (95% CI: −9.8 to −8.9) to −44.2% (95% CI: −44.4 to −43.9), reflecting a substantially more protective age distribution by 2022. This large change in PAF indicates that the compositional shift in Ghana’s age distribution contributed significantly to lowering the hypothetical national burden if all age groups attained the most favourable group’s prevalence. The age PAR also became statistically significant: shifting from −0.7% (95% CI: −3.8 to 2.5 — CI crossing zero, not statistically significant in 2008) to −2.5% (95% CI: −4.0 to −1.0 — CI excluding zero, statistically significant in 2022), indicating that by 2022, the age-related contribution to national burden was meaningfully and significantly reduced.

For the marital-status dimension, both the PAF and PAR showed only marginal changes between 2008 and 2022, as described above. The marital-status PAF remained large (>10% in absolute terms) in both years, indicating that the disproportionate burden among never-married women continued to represent a substantial source of inequality throughout the study period, with only limited change.

## 4 Discussion

This study examined differences in the estimated burden of childhood sexual violence among Ghanaian women across age cohorts and marital-status subgroups between 2008 and 2022, using four complementary WHO HEAT summary measures. The principal findings are as follows as (1) the estimated national prevalence of childhood sexual violence was lower in 2022 (6.4%) than in 2008 (8.3%) among women aged 18–29 years; (2) never-married women consistently carried a disproportionate burden, with the highest estimated prevalence in both years (11.3% in 2008; 8.4% in 2022); (3) younger cohorts (20–34 years) showed lower estimates in 2022, while some older cohorts (35–44 years) showed higher estimates, suggesting complex generational patterns; (4) the age-related PAF increased markedly in magnitude (from −9.3% to −44.2%), reflecting a more protective age distribution, and the age PAR became statistically significant in 2022; and (5) marital-status disparities showed only marginal differences between the two rounds, with the PAF remaining large (>−16%) and the PAR narrowing but losing statistical significance. These findings are discussed below in relation to the two stratifiers analysed.

The finding that never-married women consistently bear the highest burden of childhood sexual violence is consistent with evidence from comparable settings in sub-Saharan Africa. Never-married adolescent girls and young women tend to face greater exposure to coercive sexual relationships, reduced parental or household oversight during adolescence, and heightened vulnerability to transactional sex and sexual exploitation compared with their married peers (35, 36). Heise et al.’s (37) ecological framework of violence against women identifies reduced social monitoring, limited social support networks, and exposure to gender-unequal norms as key determinants of sexual violence risk among unmarried young women (37). In Ghana’s context, cultural norms that deter disclosure from never-married women, due to fears of reputational damage, rejection by future partners, or family censure, may also contribute to higher survey disclosure rates paradoxically, as never-married women may have fewer social deterrents to reporting past victimisation in anonymous survey settings than married women who fear domestic consequences (26, 38).

The persistence of this disparity across both survey rounds (never-married women maintained the highest prevalence by approximately 3–4 percentage points over married women in both years) is particularly concerning from a policy perspective. The marital-status PAF remaining large (−16 to −18%) across both years indicates that the structural source of this disparity, the elevated risk experienced by never-married young women, has not been substantially reduced over this period. Targeted interventions for never-married adolescent girls and young women, including school-based safeguarding programmes, community-based outreach through youth-serving organisations, and accessible, confidential reporting mechanisms that reduce disclosure barriers, are urgently warranted.

The observed age-cohort patterns are complex and must be interpreted carefully. Younger cohorts (20–34 years in 2022) showed lower estimated prevalence than in 2008, consistent with the hypothesis that Ghana’s strengthened child protection legal framework and improved public awareness following the Domestic Violence Act (2007) may have contributed to a reduced burden for those who experienced childhood under these improved conditions (39, 40). These findings align with evidence from Kenya, where the Sexual Offences Act (2006) was associated with improved outcomes for adolescent girls over subsequent survey rounds (41).

In contrast, the estimated prevalence increased among women aged 35–39 and 40–44 years in 2022. Several explanations are plausible. First, women in these age groups in 2022 experienced their childhoods in the 1980s and early 1990s, a period when Ghana lacked comprehensive child protection laws and public awareness of sexual violence was substantially lower (27). The higher estimates in 2022 for these cohorts may reflect growing willingness to disclose past victimisation as social norms around sexual violence disclosure have shifted. Second, older cohorts may exhibit period effects: as national discourse around gender-based violence has intensified over the 2008–2022 interval, women who previously would not have identified their childhood experiences as ‘sexual violence’ may now do so, increasing self-reported prevalence independent of true differences in exposure. This mirrors findings from South Africa, where older women were found to carry a high historical burden of unreported childhood sexual violence and showed increased disclosure in later adult surveys (42). These cohort-specific patterns demonstrate the importance of interpreting DHS-based childhood sexual violence estimates with an explicit recognition of the recall and disclosure biases that differentially affect younger versus older cohorts.

The substantial increase in the age-related PAF (from −9.3% to −44.2%) is driven primarily by the divergence in age-group estimates between 2008 and 2022, with younger cohorts declining more steeply than older cohorts and some older cohorts increasing. This results in a wider spread of age-group estimates in 2022 relative to 2008, making the hypothetical gain from equating all groups to the most favourable (lowest) level much larger in 2022. This does not necessarily indicate programme success; it may partly reflect the cohort-specific disclosure dynamics described above. Caution is therefore warranted in interpreting the PAF difference as evidence of improved equity.

The lower estimated national prevalence in 2022 (6.4%) compared with 2008 (8.3%) is consistent with evidence from other sub-Saharan African countries that have strengthened child protection systems and sexual violence legislation over comparable periods (1, 43). Similar reductions have been documented in studies of childhood sexual violence comparisons in East Africa, where legislative reforms and community-based safeguarding were associated with improved outcomes for younger cohorts (41). However, as emphasised by both international and Ghana-specific literature, the DHS relies on retrospective self-report of sensitive experiences, and observed differences in prevalence between survey rounds may reflect differences in disclosure behaviour, survey methodology, interviewer training, or social desirability bias as much as, or more than, true reductions in childhood exposure (44, 45). Ghana’s growing public discourse on gender-based violence between 2008 and 2022, amplified by social media and civil-society advocacy, may have simultaneously increased willingness to disclose (which would increase estimates) and changed what experiences are labelled as ‘sexual violence’ (which could change estimates in either direction). These interpretive complexities must be explicitly acknowledged when communicating findings to policymakers.

The WHO HEAT framework provided a structured, internationally standardised approach to quantifying the magnitude and distribution of disparities in childhood sexual violence burden across population subgroups. The four complementary summary measures, including D, R, PAF, and PAR, each contributed distinct information: D and R characterised the absolute and relative magnitude of between-group differences; PAF and PAR quantified the hypothetical burden reduction achievable if all subgroups attained the most favourable group’s prevalence. The statistical significance of PAF and PAR estimates, assessed through 95% CIs, allowed discrimination between robust and uncertain findings, for example, revealing that the age PAR was not statistically significant in 2008 but became so in 2022.

However, the HEAT approach has important limitations. It does not adjust for confounders, model causal pathways, or distinguish between-group differences that are attributable to structural inequalities versus compositional differences in population characteristics. The PAF and PAR measures, while described in terms of ‘hypothetical reduction in prevalence if disparities were eliminated,’ should not be interpreted as causal effect estimates; they assume no confounding, which cannot be verified. Future analyses using individual-level DHS data would enable multivariable logistic regression to adjust for confounders; decomposition analysis to partition observed between-group differences into explained and unexplained components; and concentration index analysis to quantify socioeconomic gradients in childhood sexual violence burden. These approaches would substantially complement the descriptive findings presented here.

## 5 Policy, practice, and research implications

The decline in sexual violence before age 18 across age and marital-status groups suggests national progress, yet persistent inequalities among never-married women and some older cohorts indicate that risks remain concentrated in specific subpopulations. These patterns necessitate targeted policy actions within Ghana’s context. The Ministry of Gender, Children, and Social Protection should prioritize expanding the reach of the Domestic Violence and Victim Support Unit (DOVVSU) to rural areas, where enforcement is often weak due to logistical and funding constraints. Additionally, integrating sexual violence prevention into the Ghana Education Service curriculum, supported by teacher training on safeguarding, can enhance school-based protection for young girls. Addressing implementation challenges, such as cultural resistance and limited budgets, will require partnerships with traditional authorities and civil-society organizations to build community trust and leverage local resources.

For practice, tailored interventions are essential to address subgroup vulnerabilities. Never-married young women, as the most affected group, require focused outreach through school-based programs and community platforms, such as youth centres, to provide education on safe relationships and reporting mechanisms. For older cohorts with rising or stable prevalence, health professionals and social workers should incorporate routine enquiry about past sexual violence in reproductive-health services, ensuring referrals to psychosocial support. Overcoming barriers like stigma and lack of trained personnel will necessitate capacity-building for frontline workers and collaboration with non-governmental organizations, faith leaders, and traditional leaders to shift harmful norms and promote safe disclosure.

## 6 Strengths and limitations

This study has several strengths. It used nationally representative GDHS data and applied the internationally validated WHO HEAT framework, providing standardised summary measures that enable comparison across countries and health indicators. The use of all four complementary HEAT measures provided a more comprehensive characterisation of disparities than any single measure alone. The comparison of two survey rounds spanning 14 years of policy reform provides an accountability perspective that single-round analysis cannot. The study has several limitations. First, and most critically, the two-round design enables comparison between two survey years but does not constitute a trend analysis in the conventional sense; the trajectory of health outcomes between 2008 and 2022 is unknown. We therefore highlight this paper as non-causal or a non-temporal change. Second, self-reported data on childhood sexual violence are subject to substantial recall bias, social desirability bias, and differential disclosure across groups and over time, which may systematically affect between-group and between-round comparisons. Third, the 2014 GDHS round was not incorporated, which limits the temporal resolution of the comparative analysis. Thus, in addressing this in future work, studies can either upload 2014 estimates to HEAT Plus or request WHO inclusion of 2014 data. Fourth, the analysis was restricted to age and marital status as stratifying dimensions due to constraints of the HEAT platform interface; other relevant equity dimensions (wealth, education, residence, region) were not examined and may carry important policy implications. Fifth, the age-stratified subgroup analysis extends beyond the 18–29-year national prevalence reference population to ages 20–49, which is a platform-determined constraint that must be carefully considered in interpretation. Finally, the HEAT framework does not adjust for confounders and does not support causal inference but is descriptive.

## 7 Conclusions

This study provides an equity-focused comparative analysis of the estimated burden of childhood sexual violence among Ghanaian women using the World Health Organization Health Equity Assessment Toolkit applied to the 2008 and 2022 Ghana Demographic and Health Surveys. Findings indicate a decline in the estimated national prevalence from 8.3% in 2008 to 6.4% in 2022, with lower estimates observed across most age and marital-status subgroups in 2022. However, these findings should be interpreted cautiously due to the cross-sectional and retrospective nature of the data, as well as potential differences in disclosure behaviours across survey periods. Despite the overall decline, never-married women consistently experienced a disproportionate burden, highlighting persistent marital-status inequalities. Age-related patterns were mixed, with younger cohorts demonstrating greater reductions while some older groups reported increased estimates, potentially reflecting generational shifts in childhood protection environments and evolving disclosure norms. The findings underscore the need for targeted prevention and survivor support interventions, particularly for never-married young women, while contributing evidence towards monitoring Ghana’s progress in achieving Sustainable Development Goal 5 on ending violence against women and girls.

## Data Availability

The dataset used in this analysis can be accessed at https://www.who.int/data/inequality-monitor/assessment_toolkit. The 2008 and 2022 Ghana DHS data are available through the MEASURE DHS programme at https://www.dhsprogram.com.

## References

[1] Cagney J Spencer C Flor L Herbert M Khalil M O'Connell E . Prevalence of sexual violence against children and age at first exposure: a global analysis by location, age, and sex (1990–2023). Lancet. (2025) 405(10492):1817–36. doi: 10.1016/S0140-6736(25)00311-3 PMC12100463 40347967

[2] World Health Organization . Violence Against Women. Geneva: WHO (2024).

[3] Devries KM Mak JYT García-Moreno C Petzold M Child JC Falder G . The global prevalence of intimate partner violence against women. Science. (2013) 340(6140):1527–8. doi: 10.1126/science.1240937 23788730

[4] Ghana Statistical Service . One in Seven Women Aged 15–49 Have Experienced Sexual Violence at Some Point in Their Lives. Accra: GSS (2024).

[5] Woldearegay H Gebretnsae H Mackey A Bigalky J Petrucka P . Understanding nature, barriers, and facilitators in addressing sexual and gender-based violence (SGBV) in conflict zones of Africa: A scoping review. BMC Public Health. (2025) 25:3583. doi: 10.1186/s12889-025-24645-5 41131508 PMC12551275

[6] Munala L Olson H Johnson C . 'If you are raped, you are like secondhand': systemic barriers to reporting sexual violence against school-aged girls in a rural community in Kenya. Sexes. (2025) 6:12. doi: 10.3390/sexes6010012 30654563

[7] Bazaanah P Ngcobo P . Shadow of justice: review on women's struggle against gender-based violence in Ghana and South Africa. SN Soc Sci. (2024) 4:126. doi: 10.1007/s43545-024-00926-5 30311153

[8] Borumandnia N Khadembashi N Tabatabaei M Alavi Majd H . The prevalence rate of sexual violence worldwide: a trend analysis. BMC Public Health. (2020) 20:1835. doi: 10.1186/s12889-020-09926-5 33256669 PMC7706187

[9] Xianguo Q Hui C Xin S Jing F Zijian W Zhenyu N . The prevalence of sexual violence against African women: a systematic review and meta-analysis. Afr Health Sci. (2023) 23(3):117–27. doi: 10.4314/ahs.v23i3.15 PMC10862609 38357142

[10] Ghana Statistical Service Ghana Health Service ICF . Ghana Demographic and Health Survey 2022. Accra (2023).

[11] Ghana Statistical Service Ghana Health Service ICF . Ghana Demographic and Health Survey 2008. Accra (2009).

[12] UNICEF . Fulfilling the Commitment to Child Protection in Ghana. New York: UNICEF (2021).

[13] Fielding-Miller R McDougal L Frost E Masuku S Shabalala F . Association between sexual violence and depression is mediated by perceived social support among female university students in Eswatini. BMC Public Health. (2024) 24:2526. doi: 10.1186/s12889-024-20040-8 39289704 PMC11406860

[14] Lee KS Wolke D Bärnighausen T Ouermi L Bountogo M Harling G . Sexual victimisation, peer victimisation, and mental health outcomes among adolescents in Burkina Faso: a prospective cohort study. Lancet Psychiatry. (2024) 11(2):134–42. doi: 10.1016/S2215-0366(23)00399-1 PMC11932973 38245018

[15] Johansson F Edlund K Sundgot-Borgen J Björklund C Côté P Onell C . Sexual harassment, sexual violence and subsequent depression and anxiety symptoms among Swedish university students: a cohort study. Soc Psychiatry Psychiatr Epidemiol. (2024) 59(12):2313–22. doi: 10.1007/s00127-024-02688-0 PMC11522111 38926188

[16] Woldie SA Walker G Bergman S Diemer K Block K Armstrong G . The association between sexual violence and mental disorders among women victim-survivors in sub-Saharan Africa: a systematic review and meta-analysis. BMJ Glob Health. (2025) 10(3). doi: 10.1136/bmjgh-2024-017962 PMC11931965 40122529

[17] Geli J Harmel C Liautard M Deguette C Alcaraz E Dang C . Contamination by sexually transmitted infections following sexual violence: A retrospective study at the Forensic Unit of Hôtel-Dieu, Paris, France. Forensic Sci Int. (2025) 378:112690. doi: 10.1016/j.forsciint.2025.112690 41161034

[18] Amone-P'Olak K Lekhutlile TM Ovuga E Abbott RA Meiser-Stedman R Stewart DG . Sexual violence and general functioning among formerly abducted girls in Northern Uganda: the mediating roles of stigma and community relations - the WAYS study. BMC Public Health. (2016) 16(1):64. doi: 10.1186/s12889-016-2735-4 26801899 PMC4722696

[19] Schnittker J . What makes sexual violence different? Comparing the effects of sexual and non-sexual violence on psychological distress. SSM Ment Health. (2022) 2:100115. doi: 10.1016/j.ssmmh.2022.100115 38826717

[20] Ahinkorah BO . Intimate partner violence against adolescent girls and young women and its association with miscarriages, stillbirths and induced abortions in sub-Saharan Africa: Evidence from demographic and health surveys. SSM Popul Health. (2021) 13:100730. doi: 10.1016/j.ssmph.2021.100730 33511264 PMC7815812

[21] Tenkorang E . Sexual violence at first intercourse and subsequent sexual practices among Ghanaian women. Violence Against Women. (2021) 27:1352–74.

[22] Parliament of Ghana . Domestic Violence Act 732. Accra: Parliament of Ghana (2007).

[23] Mabasa C Mukoma G Manganye B . Gender-based violence and women's wellness in rural South Africa. Int J Environ Res Public Health. (2025) 22:887. doi: 10.3390/ijerph22060887 40566314 PMC12193001

[24] Ullman S O'Callaghan E Shepp V Harris C . Reasons for and experiences of sexual assault nondisclosure in a diverse community sample. J Fam Violence. (2020) 35:839–51. doi: 10.1007/s10896-020-00141-9 33746358 PMC7967004

[25] Idle A . Legal frameworks for addressing gender-based violence in east africa: progress and gaps. (2025), 770–97.

[26] Boakye KE Asare-Doku W Deliege A Bansal A Khan MR . Context and disclosure of female child sexual abuse: analysis of victims case reports in Ghana. Child Youth Serv Rev. (2025) 179:108601. doi: 10.1016/j.childyouth.2025.108601

[27] Lu M Owusu-Addo E Yoshikawa M Mensah H Fry D . Understanding law enforcement interventions for the response to and prevention of sexual exploitation and abuse against children and women in Ghana: Findings from a realist review. Aggress Violent Behav. (2025) 83:102068. doi: 10.1016/j.avb.2025.102068

[28] World Health Organization . Health Equity Assessment Toolkit (Heat): Software for Exploring and Comparing Health Inequalities in Countries. Geneva: WHO (2023). 10.1186/s12874-016-0229-9 PMC5069829 27760520

[29] Okyere J Azure SA Budu E Mensah F Ahinkorah BO Ameyaw EK . Trends and inequalities in children aged 6-59 months who received Vitamin A supplementation: evidence from the 2003, 2008 and 2014 Ghana Demographic and Health Survey. Trop Med Health. (2022) 50(1):99. doi: 10.1186/s41182-022-00488-3 36578095 PMC9795595

[30] Shibre G Zegeye B Haidar J . Extent of and trends in inequalities in child stunting in Sierra Leone from 2005 to 2013. Int J Equity Health. (2020) 19:88. 32503547 10.1186/s12939-020-01212-5 PMC7275402

[31] Osborne A Bangura C Sesay U . Trends and inequalities of minimum dietary diversity among children aged 6–23 months in Sierra Leone, 2013–2019. (2025), 1–9. doi: 10.1186/s12889-025-22891-1 PMC12085039 40382538

[32] ICF International . Demographic and Health Survey Methodology. Calverton MD: ICF (2012).

[33] Jewkes R Fulu E Roselli T Garcia-Moreno C . Prevalence of and factors associated with non-partner rape perpetration: findings from the UN Multi-country Cross-sectional Study on Men and Violence in Asia and the Pacific. Lancet Glob Health. (2013) 1:e208–18. doi: 10.1016/s2214-109x(13)70069-x 25104346

[34] Oduro G Swartz S Arnot M . Understanding gender inequality and girls' experiences of violence in developing countries. Int J Educ Dev. (2012) 32:752–60.

[35] Ramamurthy D Lonimath A Kannan S Khazi M . Behind closed doors: national survey insights on sexual violence among ever-married women in India. Discov Public Health. (2025) 22:266. doi: 10.1186/s12982-025-00667-7 38164791

[36] UNICEF . Overcoming the Challenges of Education in Uganda. New York: UNICEF (2024).

[37] Heise L Greene ME Opper N Stavropoulou M Harper C Nascimento M . Gender inequality and restrictive gender norms: framing the challenges to health. Lancet. (2019) 393(10189):2440–54. doi: 10.1016/S0140-6736(19)30652-X 31155275

[38] Chavula MP Matenga TFL Halwiindi H Hamooya C Sichula N Jones DL . Factors shaping responsiveness towards sexual gender-based violence during the COVID-19 Pandemic in Africa: A systematic review. Cogent Public Health. (2023) 10(1):2234600. doi: 10.1080/27707571.2023.2234600

[39] Gamble-Payne K Freeman J . Systems for the safety and protection of children. In: Protecting the World's Children (2025).

[40] Seedat S Scott KM Angermeyer MC Berglund P Bromet EJ Brugha TS . Cross-national associations between gender and mental disorders in the World Health Organization World Mental Health Surveys. Arch Gen Psychiatry. (2009) 66(7):785–95. doi: 10.1001/archgenpsychiatry.2009.36 PMC2810067 19581570

[41] Wangamati C Yegon G Sundby J Prince R . Sexualised violence against children: a review of laws and policies in Kenya. Sex Reprod Health Matters. (2019) 27:1586815. doi: 10.1080/26410397.2019.1586815 31533564 PMC7887981

[42] Mabasa C Mukoma G Manganye B . 'We report to traditional leaders, but patriarchy means we rarely win the case'. Int J Environ Res Public Health. (2025) 22:887. doi: 10.3390/ijerph22060887 40566314 PMC12193001

[43] García-Moreno C Zimmerman C Morris-Gehring A Heise L Amin A Abrahams N . Addressing violence against women: a call to action. Lancet. (2015) 385(9978):1685–95. doi: 10.1016/S0140-6736(14)61830-4 25467579

[44] Hahn J McCormick M Silverman J Robinson E Koenen K . Examining the impact of exposure to discrimination intersectionality on self-rated health outcomes among a diverse national sample. PloS One. (2021) 16:e0254935. 34297760

[45] WHO LSHTM MRC . Preventing Intimate Partner and Sexual Violence Against Women: Taking Action and Generating Evidence. Geneva: WHO (2010). 10.1136/ip.2010.029629 20921563

